# Selective ATM inhibition augments radiation-induced inflammatory signaling and cancer cell death

**DOI:** 10.18632/aging.204487

**Published:** 2023-01-17

**Authors:** Li-Ya Chiu, Qing Sun, Frank T. Zenke, Andree Blaukat, Lyubomir T. Vassilev

**Affiliations:** 1 Translational Innovation Platform Oncology and Immuno-Oncology, EMD Serono, Billerica, MA 01821, USA; 2 Translational Innovation Platform Oncology and Immuno-Oncology, The Healthcare Business of Merck KGaA, Darmstadt, Hesse, Germany

**Keywords:** ATM, radiation therapy, cell cycle, micronuclei, STING

## Abstract

Over half of all cancer patients undergo radiation therapy but there is an unmet need for more efficacious combination strategies with molecular targeted drugs. DNA damage response has emerged as an important intervention point for improving anti-tumor effects of radiation and several inhibitors are currently in development. Ataxia telangiectasia mutated (ATM) kinase is a key regulator of cellular response to DNA double strand breaks and a potential target for radiosensitization. We recently reported two new potent and selective ATM inhibitors, M3541 and M4076, that effectively sensitize cancer cells to radiation and regress human xenografts in clinically relevant animal models. Here, we dive deeper into the cellular events in irradiated cancer cells exposed to ATM inhibitors. Suppression of ATM activity inhibited radiation-induced ATM signaling and abrogated G1 checkpoint activation resulting in enhanced cell death. Our data indicated that entry into mitosis with gross structural abnormalities in multiple chromosomes is the main mechanism behind the increased cell killing. Misalignment and mis-segregation led to formation of multiple micronuclei and robust activation of the interferon response and inflammatory signaling via the cGAS/STING/TBK1 pathway. Cancer cells exposed to radiation in the presence of M3541 were more susceptible to killing in co-culture with NK cells from healthy donors. In addition, strong upregulation of PD-L1 expression was observed in the surviving irradiated cancer cells exposed to M3541. Simultaneous activation of the STING pathway and PD-L1 suggested that combination of radiation, ATM inhibitors and PD-L1 targeted therapy may offer a novel approach to radio-immunotherapy of locally advanced tumors.

## INTRODUCTION

Ionizing radiation (IR) is an important treatment modality and nearly 60% of all cancer patients undergo radiation therapy in the course of their disease [1, 2]. Radiation induced antitumor activity is primarily driven by the DNA damage response (DDR), a large network of cellular pathways evolved to detect and repair DNA lesions and protect genome integrity [3, 4]. Radiation induced double strand breaks (DSB) are the most difficult to repair and if left unrepaired can lead to genome instability and ultimately cell death.

Ataxia-Telangiectasia Mutated (ATM) kinase, acts as an upstream signaling node in the detection and repair of DSBs [5, 6]. Activated ATM phosphorylates H2AX on serine 139 known as γH2AX [7] to initiate and sustain chromatin based signaling at damaged sites [8]. It simultaneously activates multiple pathways to control DSB repair and the cell cycle checkpoint machinery [5, 9]. ATM coordinates the response to DSBs by activating checkpoint kinase 2 (CHK2), the p53 tumor suppressor and its downstream transcriptional target p21, resulting in G1/S and G2/M arrest to prevent cells from entering replication and mitosis until DSBs are successfully repaired [10–12]. In addition to its role as a key controller of the cell cycle checkpoints, ATM plays an important role in DSB repair. Hence, the presence of functional ATM determines the ability of cells to elicit proper DDR and represents one of the key determinants of the cellular sensitivity to radiation [13]. The importance of ATM activity in DSB repair is illustrated by the fact that cells from Ataxia Telangiectasias (AT) patients exhibit marked chromosomal instability and sensitivity to radiation and other DNA damaging agents [14].

In addition to directly targeting cancer cell cycle and viability, IR can enhance anti-tumor immunity through the activation of interferon (IFN) signaling [15, 16]. Type I IFN signaling can bridge innate and adaptive immune responses by facilitating dendritic cell maturation and infiltration into tumors, crucial for priming and stimulation of tumor-specific T cell activity. The cytoplasmic nucleic acid sensing pathway - cyclic GMP-AMP synthase (cGAS) and stimulator of interferon genes (STING) are key mediators in the regulation of type I IFN production [17–19]. DNA DSB damaging agents, including radiation, could promote formation of micronuclei with compromised lamina allowing cytosolic exposure of dsDNA, a substrate necessary for activation of cGAS/STING pathway, IFN signaling and inflammatory response in cancer cells [20–24]. These studies suggested that the cGAS/STING pathway plays a key role in the immune response of irradiated cancer cells.

Despite the continuous progress made in the targeted delivery of radiation, new combination approaches to improve its efficacy and safety are highly sought-after. In the last decade, targeting key regulatory nodes in DDR have emerged as an area of high interest and promising combination strategies have evolved together with new agents. Several new ATM inhibitors have been reported and shown to enhance the efficacy of radiation, PARP inhibitors and chemotherapy [25–29]. However, our understanding of how inhibition of ATM activity impacts radiation-induced cell death, and the immune response is still evolving. Using the recently reported potent and selective ATM inhibitors, M3541 and M4076 [30], we show that suppression of ATM catalytic activity potentiates radiation-induced cancer cell killing by lowering the cell cycle checkpoint barrier to allow mitotic entry of cancer cells with severely damaged chromosomes from unrepaired DSBs. We further demonstrate that misalignment and mis-segregation during aberrant mitosis leads to pronounced micronucleation, a powerful mechanism for activation of cGAS/STING induced inflammatory signaling in irradiated cancer cells exposed to the ATM inhibitors.

## MATERIALS AND METHODS

### Cell lines and reagents

All used cancer cell lines were obtained from American Type Culture Collection (ATCC) if not indicated otherwise. A549 and A375 cells were cultured in Dulbecco’s Modified Eagle Media (DMEM). HeLa, HT1080 and RKO cells were maintained in Minimum Essential Media (MEM) and H1299 cells were cultured in RPMI 1640 media. A549 NucLight Red (4491) and HeLa NucLight Green (4490) cells were purchased from Essen Bioscience and cultured according to the manufacturer’s protocols. HeLa-GFP-H2B cells were provided by Geoffrey Wahl (The Salk Institute for Biological Studies) [31]. Both cell lines were cultured in DMEM. All cells were supplemented with 10% fetal bovine serum (FBS, Corning) and incubated at 37° C in the presence of 5% CO_2_. ATM inhibitors M3541 and M4076 were synthesized at Merck KGaA, Darmstadt, Germany [30]. MRT67307 was purchased from MilliporeSigma. RO-3306, AZD7762 were obtained from SelleckChem and colcemid was purchased from Roche Diagnostics. All compounds were dissolved in DMSO to prepare a 10 mM stock and stored at -80° C.

### Western blotting

Cell lysates were collected by directly lysing cells in 2x Laemmli buffer (120 mM Tris-HCl, pH 6.8, 4% sodium dodecyl sulfate, 20% glycerol and) supplemented with protease and phosphatase inhibitors cocktail (Roche Diagnostics). Samples were loaded on NuPAGE 4-12% gradient Bis-Tris Gels and transferred to nitrocellulose membranes. Membranes were incubated with different antibodies, including Abcam: p-ATM (ab81292), ATM (ab199726); Cell Signaling Technology: p-p53 (9284), p53 (48818), p-CHK2 (2197), CHK2 (3440), p21 (2947), p-ATR (58014), ATR (13914), p-CHK1 (12302), p-CHK1 (2348), CHK1 (2360), GAPDH (3683), p-STING (19781), STING (13647), p-TBK1 (5483), TBK1 (3013), p-IRF3 (4947), IRF3 (14302), p-RELA (3033), RELA (8242), IκBα (9242), p-STAT (9167), STAT (9176), PD-L1 (13684), Invitrogen Vinculin (700062) and proceeded to chemiluminescent detection according to the manufacturer instruction. SuperSignal™ West Pico Chemiluminescent Substrate or West Femto Maximum Sensitivity Substrate (ThermoFisher) were used and analyzed by ImageQuant LAS 4000 (GE Healthcare).

### Meso scale discovery (MSD) assays

For the detection of p-ATM, cells were treated and lysed in Tris lysis buffer (Meso Scale Discovery, MSD, R60TX-3) supplemented with protease and phosphatase inhibitors. Briefly, multi-array 96 well plate MSD plates (MSD, L15XA-3) were coated with the capture antibody against phosphorylated ATM (Abcam, ab208775) at 4° C overnight. The next day, plates were washed with Tris Wash Buffer (MSD, R61TX-2), incubated with 3% Blocker A (MSD, R93BA-4) for 1 hour. Equal amount of protein lysates was added and incubated for 2 hours, washed and sequentially incubated with two detection antibodies, ATM (Santa Cruz biotechnology, Sc1356630) and MSD SULFO-TAG labeled anti-mouse (MSD, R32AC-1) for 1 hour. The plates were read using a Sector 1300 imager (MSD). The values were normalized to the DMSO controls, and the background-corrected values were analyzed by setting the mean value of IR-treated samples to 100% and transforming the values measured for the IR+ATM inhibitor-treated samples to percentages. For determining cytokine/chemokine secretion profile in culture media, MSD Assay kits (K15067M, K15053K, F21ZN) were used. Standard and samples were measured and performed according to the manufacturer's protocol. Data analysis was done using the Discovery workbench software (MSD). Protein concentrations were further normalized to total cell number for each treatment condition.

### Cell cycle analysis

Cells were pre-treated with 1 μM ATM inhibitor 1 hour prior to IR (5Gy) and 24 h later pulsed with 10 μM BrdU for 1 h, fixed in ice-cold 70% ethanol and left at -20° C overnight. The next day, cells were washed once with 2% FBS in PBS, and incubated with 2 N HCl and 0.5% Triton X-100 at RT for 1 h, neutralized with 0.1 M sodium tetraborate, pH 8.5 and then stained with FITC-conjugated anti-BrdU antibody (BD Bioscience, 347583) followed by 7-Amino-Actinomycin D (7-AAD) staining (BD Bioscience, 559925). Cell cycle profiles were analyzed by BD FACS Canto flow cytometer and data were processed with the FlowJo software.

### IncuCyte live-cell imaging

A549 NucLight and HeLa NucLight cells were seeded in 96-well plates and incubated at 37° C with 5% CO_2_ overnight. The next day, cells were pre-treated with M3541 for 1 hour followed by irradiation (5 Gy). IncuCyte™ Cytotox Red reagent (Essen Bioscience, 4632) was added to the medium to detect dead cells in real-time. Images were taken every 2 h over a 6-day period using an IncuCyte ZOOM instrument (Essen Bioscience). Cell growth curves were plotted as number of fluorescent green nuclei over time. Relative cell death is determined by the counts of IncuCyte™ Cytotox Red labelled cells normalized to total number of green nuclei per well. To monitor mitosis of HeLa GFP-H2B cells they were treated and imaged every 10 min with 20x objective for 7 days. ImageJ (National Institutes of Health) was used to track and analyze the movement of mitotic chromosomes individually.

### Metaphase spreads and spectral karyotyping analysis

Cells were seeded and cultured overnight then pre-treated with M3541 (1 μM) for 1 h before IR (5 Gy). After 24 hours, colcemid (0.1 μg/ml) was added and cells incubated for additional 4 h. Mitotic cells were collected by shake off and gently mixed in a hypotonic KCl buffer (0.57%) for 30 min at 37° C and fixed in acetic methanol (1:3, v/v) solution and dropped onto slides. Slides were stained with Giemsa. 50 metaphase spreads per treatment were scored for chromosomal aberrations. For spectral karyotyping (SKY) analysis, fixed cells were processed as described elsewhere [32]. Ten metaphase spreads were analyzed per treatment condition.

### Immunofluorescence

Immunofluorescence studies were performed as previously described [30]. Briefly, cells were seeded in glass chamber slides (MilliporeSigma, PEZGS0816). Cells were then treated and fixed in fixing solution (1% paraformaldehyde, 2% Sucrose in PBS) for 15 min at room temperature followed by ice-cold methanol at -20° C for 30 min, methanol/acetone at -20° C for 20 minutes. Cells were blocked and incubated with γH2AX (Cell Signaling Technology, 9718) and alpha-tubulin (Abcam, 7291) overnight at 4° C, washed and incubated with secondary antibodies for 1hr. Cells were then counterstained with DAPI (Invitrogen, D1306) and mounted onto glass slides with ProLong® Gold Antifade Mountant (Invitrogen, P36934). Images were taken using a fluorescence microscopy Zeiss MIC-074. γH2AX foci were analyzed by ImageJ.

### Quantitative PCR

Total RNA was isolated by RNeasy Mini Kit (Qiagen, 74104) with on column DNase digestion (Qiagen, 79254) following the instructions of the manufacturer. 2 μg RNA was reverse transcribed using SuperScript VILO master mix (Thermo Fisher Scientific, 11755050) as described by manufacturer. cDNA was then diluted, and quantitative PCR was performed using TaqMan Fast Advanced Master Mix and a QuantStudio 7 Flex Real-Time PCR instrument (Applied Biosystems). Relative fold change (-ddCt) gene expression was normalized to its GAPDH and DMSO treated samples. Taqman probes are listed in the Supplementary Information.

### NK cell isolation and co-culture experiments

Human natural killer cells were isolated from peripheral blood mononuclear cells (PBMCs) using NK cell isolation kit (Miltenyi Biotechnology, 130-092-657) according to the manufacturer’s instruction. Human PBMCs were isolated from Buffy coats obtained from healthy volunteers provided by the New York Blood Center according to the protocols described elsewhere. The condition media were collected from cancer cells treated for 7 days, centrifuged at 1500 g for 10 mins and filtered by 0.22 μm Millipore filters. Treated A549 NucLight Red cells were seeded and incubated in conditioned media overnight. Fresh isolated NK cells were added at an NK to Cancer cell ratio 10:1 with or without recombinant human IL-2 (R&D systems, 202-IL-010). Cells were imaged by IncuCyte every 2 hours for 7 days and the total number of red nuclei counts per well were analyzed to determine NK cell cytotoxic activity.

### Statistical analyses

All statistical tests were performed with GraphPad PRISM v. 8.0 using unpaired T-test. P values <0.05 were considered statistically significant. Significance values: *p < 0.05, **p < 0.01, and ***p < 0.001. NS stands for non-significant (p > 0.05). All experiments were conducted independently at least three times, unless indicated otherwise, and representative data is shown as mean ± SD or SEM where indicated.

## RESULTS

### M3541 suppresses radiation-induced DSB repair and modifies cell cycle checkpoint control in cancer cells

**Figure 1:**
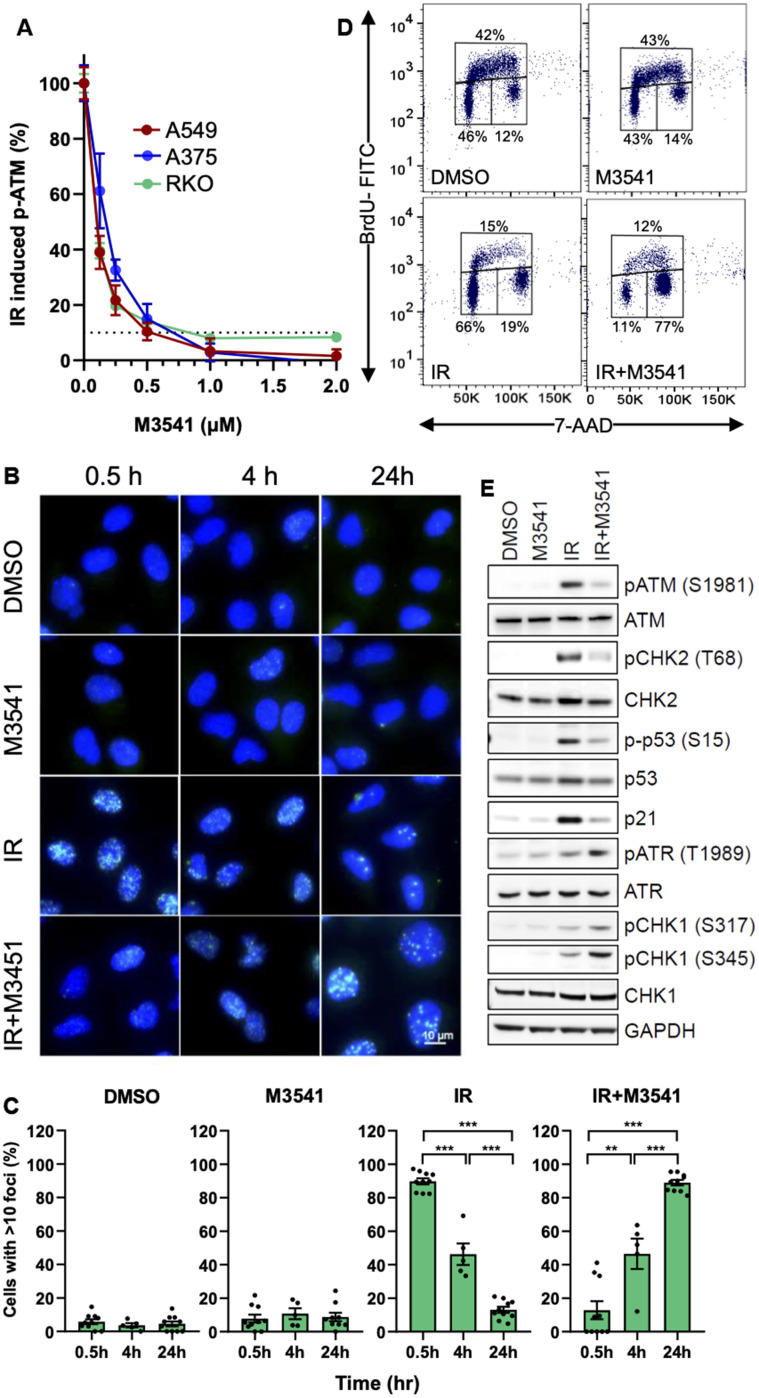
**M3541 suppresses DNA DSB repair and modulates cellular response to radiation.** (**A**). Dose-dependent inhibition of ATM autophosphorylation by M3541 in response to radiation. Proliferating A549, A375 and RKO cells were exposed to 5Gy IR to increasing concentrations of M3541 for 1 hour. Cell lysates were collected and ATM phosphorylation at serine 1981 was determined by MSD. Dashed line indicated 90% inhibition. (**B**) Analysis of γH2AX foci in A549 cells following exposure to DMSO, 1μM M3541, 5Gy IR, or combination of both for 0.5, 4 and 24 h by immunofluorescence. DAPI was used for nuclear counterstain and images were taken at 63x magnification. Scale bar is 10 μm. (**C**) Quantification of γH2AX foci was done by counting the number of cells with >10 foci in images from (**C**) after indicated times post radiation. (**D**) BrdU cell cycle analyses of A549 cells exposed to DMSO, M3541, IR or IR+M3541 for 24 h by flow cytometry. The percentage of cells in each phase was calculated and representative data are shown. (**E**) Western blot analysis of the effect of M3541 on IR-induced ATM and ATR signaling in A549 cell lysates prepared 6 h post IR. Representative images are shown.

M3541 and M4076 were recently reported as highly potent ATM kinase inhibitors in cancer cells and animal cancer models [30]. They were used as selective probes to investigate ATM‘s role in DDR and the potential therapeutic implications. First, we determined the optimal concentration of M3541 that effectively inhibits ATM function in cancer cells exposed to 5 Gy ionizing radiation by measuring ATM autophosphorylation site at serine 1981, a widely used marker for ATM activation [33]. We chose three cancer cell lines expressing wild-type ATM and p53 protein (A549, A375 and RKO). The concentration-dependent reduction of ATM phosphorylation showed that M3541 suppressed ATM activity. In all three lines, 1 μM M3541 caused over 90% ATM inhibition when compared to IR alone (Figure 1A). Therefore, we used this fixed concentration in most of our cellular assays.

In addition to ATM autophosphorylation, H2AX is one of the main ATM phosphorylation targets in response to DNA damage and γH2AX is a widely used marker for unrepaired DSBs [7, 34]. We evaluated the effect of the ATM inhibitor on IR-induced γH2AX foci in A549 cells within 24 hours post-radiation. M3541 alone had minimal effect on the number of γH2AX foci. IR produced a robust H2AX phosphorylation at an earlier time point of 30 mins. The number of γH2AX foci decreased significantly after 4 h and came close to basal levels 24 h post radiation (Figure 1B, 1C). Upon co-treatment with M3541, the γH2AX foci were dramatically reduced at 30 minutes compared to IR alone, indicating that M3541 effectively inhibited ATM-mediated H2AX phosphorylation. However, the number of γH2AX foci increased with time and at 24 h were substantially higher (>10 γH2AX foci in ~90% of the cell population) than IR or M3541 alone (~10%). The fraction of cells with >10 foci reached >80%, like the 30 min timepoint of the IR only treatment at which time most of the IR-induced DSBs were still unrepaired. Similar results were obtained by treating cells with the ATM inhibitor, M4076 (Supplementary Figure 1A–1C). The increased number of γH2AX foci in the presence of the ATM inhibitors is a likely consequence of a previously reported compensatory activation of DNA-dependent protein kinase (DNA-PK) in absence of ATM activity [7]. Indeed, treatment of A549 cells with the selective DNA-PK inhibitor peposertib (M3814) [35, 36] suppressed γH2AX levels in cells co-treated with IR+M3541 (Supplementary Figure 1D) for 30 minutes confirming that DNA-PK phosphorylated H2AX in the absence of ATM activity. Our experimental data indicated that specific inhibition of ATM catalytic activity by M3541 or M4076 extends the life of IR-induced DSBs in cancer cells.

Following DNA damage, ATM-dependent phosphorylation of p53 and CHK2 plays an important role in the cell cycle checkpoint response to DNA damage [10, 37]. Therefore, we investigated the effects of M3541 on cell cycle progression after radiation. A549 cells were exposed to DMSO, M3541 (1 μM), IR (5 Gy) or combination of IR+M3541 for 24 h and BrdU cell cycle analysis was performed. Compared with the vehicle group, M3541 alone did not affect cell cycle distribution (Figure 1D). IR increased mainly the cell population undergoing G1/S arrest and slightly G2/M phase arrest, with a concurrent decrease in S phase cells, indicating that both G1/S and G2/M checkpoints were activated upon IR treatment. When cells were irradiated in the presence of M3541, they underwent a cell cycle shift from G1 to predominantly G2/M phase arrest (Figure 1D). The G2/M phase accumulation indicated stronger activation of a G2/M checkpoint, likely reflecting the involvement of the ATR-CHK1 pathway in response to the increased number of unrepaired DSBs in the S phase cell population.

We then analyzed the changes in DNA damage signaling following M3541 treatment at a protein level. Western blotting confirmed that M3541 suppressed IR-induced ATM activation and its downstream signaling manifested by phospho-CHK2^T68^ and phospho-p53^Ser15^ increase as well as total p53 stabilization. The IR induced p53 transcriptional target p21 was also inhibited by M3541. In contrast, addition of M3541 increased the levels of phospho- ATR^T1989^, phospho-CHK1^S317^ and phospho-CHK1^S345^. We further probed the role of CHK1 in the enhanced G2/M arrest by a specific CHK1/2 inhibitor, AZD7762. Co-treatment of A549 cells reverted the IR+M3541 induced additional G2/M accumulation (Supplementary Figure 1E). The G1 phase inhibitory effect of M3541 was not limited to A549 cells, as similar cell cycle inhibitory effects were seen in two other p53 wild-type cancer cell lines, A375 and HT1080 (Supplementary Figure 1F). Taken together, these results indicate that M3541 inhibited ATM functions in both DSB repair and cell cycle checkpoint control in irradiated cancer cells and modified ATM-dependent checkpoint response by shifting cell cycle arrest to predominantly G2/M.

### M3541 enhances radiation induced cancer cell death by disrupting mitosis

**Figure 2:**
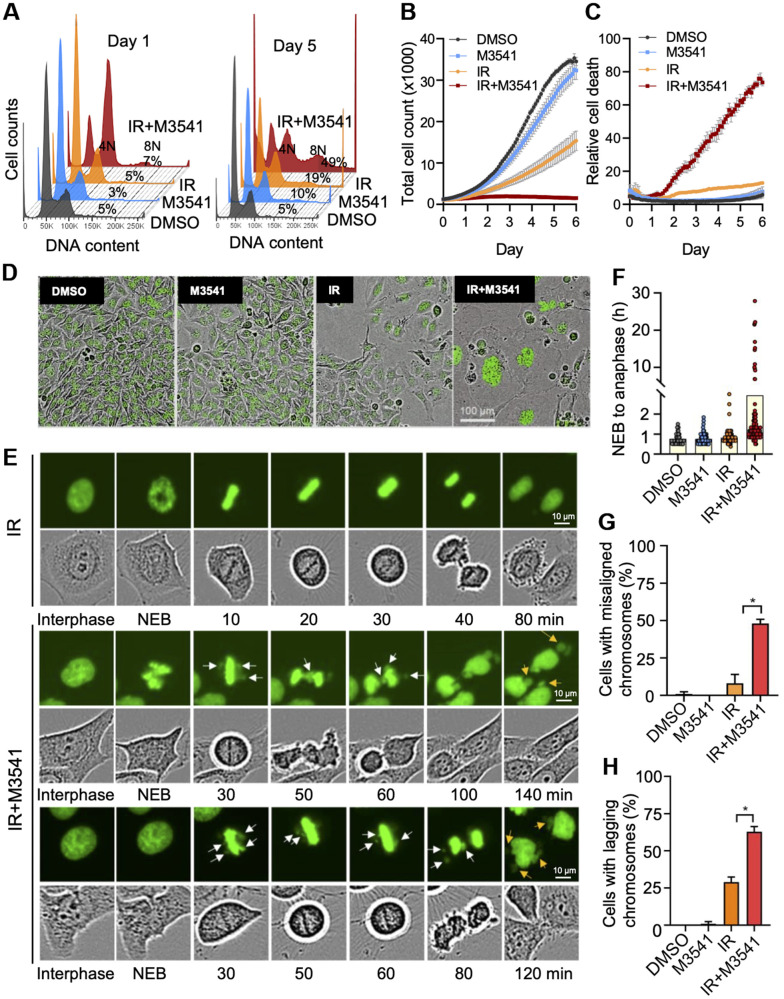
**M3541 enhances radiation-induced cancer cells death by disrupting mitosis.** (**A**) Cell cycle analysis of 7-AAD labeled HeLa cells exposed to DMSO, M3541, IR or IR+M3541 for 24 h and 5 days. Percentage of cells with more than 4N DNA content were calculated and shown from representative experiments. (**B**) Cell growth and (**C**) relative cell death in proliferating HeLa NucLight Green cells exposed to DMSO, M3541, IR or combination of IR+M3541 in the presence of Cytotox Red reagent. The cells were imaged every 2 h for 5 days by IncuCyte. Growth curves were built from the number of green-fluorescent nuclei at each time point. Relative cell death was determined as Cytotox Red positive counts normalized to green nuclei counts. Data are shown as mean ± SEM. (**D**) Representative still images from (**B**) were extracted from time-lapse videos taken by IncuCyte and shown at Day 5 post IR exposure. (**E**) Live cell imaging of HeLa cells expressing GFP tagged H2B exposed to IR or IR+M3541 by IncuCyte with a 20X objective. Individual cells were tracked in time-lapse videos and analyzed by ImageJ. Representative phase contrast and GFP images are shown. White arrows indicate chromosomal material that fails to align at the metaphase plate and lagging chromosomes. Orange arrows point to lagging chromosomes. (**F**) The lengths of time from nuclear envelop breakdown (NEB) to anaphase onset in HeLa GFP-H2B determined as in (**E**). (**G**) The percentage of metaphase cells with misaligned chromosomes and (**H**) the percentage of anaphase cells with lagging chromosomes were determined from time-lapse live imaging videos as in (**E**) Total 100 mitotic cells per condition were tracked from 2 independent experiments.

We next examined the long-term cell fate after IR+M3541 induced G2/M phase arrest. DNA content analysis of HeLa (Figure 2A) after 7-AAD staining revealed a time-dependent increase in the population of polyploid (8N) and aneuploid cells (> 4N) in the combined IR+M3541 treatment. We further assessed the impact of M3541 on cell growth and morphology by live cell imaging. HeLa NucLight cells with green fluorescent nuclei due to GFP expression in the nuclear lamina, were used in the presence of Cytotox Red dye to label dying cells. M3541 had a minimal and IR alone moderate effects on cell growth (Figure 2B). The combination of IR and M3541 strongly inhibited cell growth (Figure 2B) and dramatically increased cell death compared to IR or M3541 alone (Figure 2C). A substantial fraction of the cells surviving the 5-day combination treatment was found with enlarged cell size and multiple nuclei (Figure 2D), suggestive of abnormal cell division. We next monitored mitotic chromosomes dynamics using engineered HeLa cells stably expressing GFP labeled histone H2B [31]. Time lapse imaging and analysis revealed that the duration of nuclear envelope breakdown (NEB) to anaphase onset increased from 40-60 min in the DMSO, M3541, and IR treated cells to 72-840 min in IR+M3541 treated cells (Figure 2E, 2F), indicating that a subset of IR+M3541 treated cells spent a considerable amount of time in metaphase. Consistent with this observation, we found significant increases of misaligned chromosomes in the cells treated with IR+M3541 (48%) vs. IR-only cells (8%) (Figure 2G). The radiation exposure caused an increased number of lagging chromosomes in anaphase (29%). This effect was much more pronounced in the combined IR+M3541 treatment (63%) (Figure 2H). A failure in chromosome alignment and segregation produced daughter cells with abnormal nuclear structure, micronuclei formation and cell death during or after mitosis (Figure 2C–2E). Collectively, these results suggest that selective ATM inhibition by M3541 significantly enhances radiation induced misalignment, mis-segregation, and cell death.

### Exposure to ATM inhibitor resulted in gross chromosomal aberrations and intense micronucleation in irradiated cancer cells

**Figure 3:**
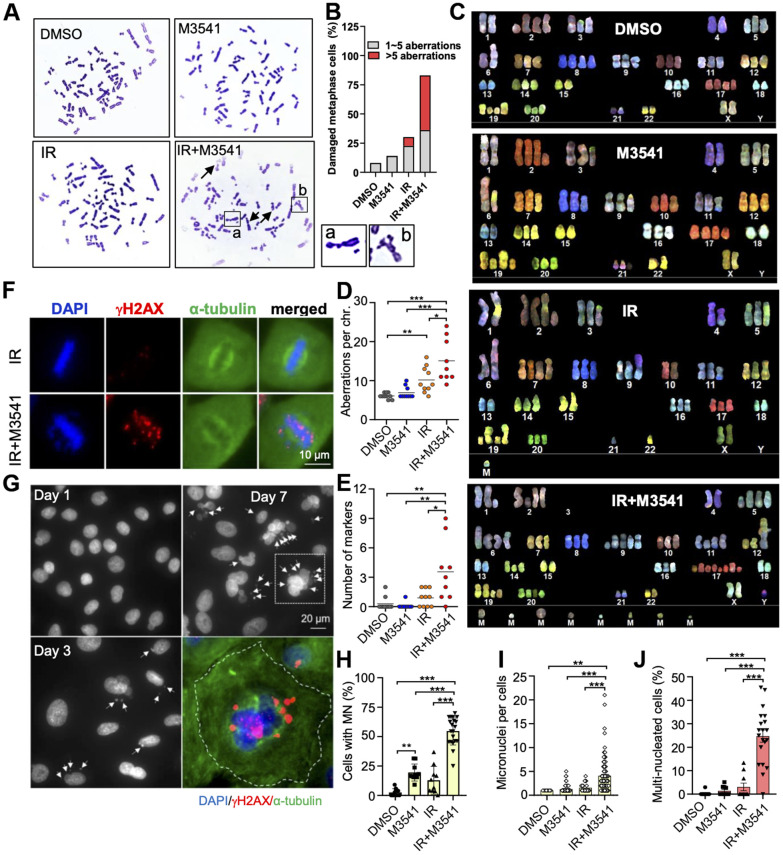
**Exposure to M3541 leads to gross chromosomal aberrations and intense micronucleation in irradiated cancer cells.** (**A**) Metaphase spread images of A549 cells exposed to DMSO, M3541, IR or combination of IR+M3541 for 24 h. Examples of chromosome aberrations are shown from representative images. (**B**) Quantification of chromosome aberrations including broken and deformed chromosomes from (**A**). (**C**) Spectral karyotyping (SKY) analyses of A549 exposed as above. (**D**) Quantification of chromosome aberrations, including insertion, duplication, deletion, translocation and chromatid breaks. (**E**) Number of unidentifiable chromosome markers (M) in each metaphase spread subjected to SKY analyses. (**F**) Mitotic cells imaged by immunofluorescence staining of DNA (DAPI), mitotic spindle (anti-α-tubulin) and unrepaired DSBs (anti-γH2AX). Proliferating A549 cells were exposed to IR or IR+M3541 enriched in G2 phase cells by the CDK1 inhibitor RO-3306 for 16h and released for 45 min to enrich in mitotic cells. (**G**) Micronuclei imaging analysis of A549 cells exposed to IR+M3541 for 1, 3 and 7 days by immunofluorescence with DAPI staining. Representative images are shown. Micronuclei were co-stained for γH2AX in the zoomed image. (**H**) Quantification of the percentage of cells with micronuclei in A549 cells exposed to DMSO, M3541, IR or both for 7 days. Data represent mean±SEM (N=2). (**I**) Number of micronuclei per cells and (**J**) Percentage of cells with > 2 nuclei. Data represent mean±SEM (N=2).

Persistent DNA damage can cause toxic chromosomal aberrations [4]. We examined the structural integrity of metaphase chromosomes in A549 cells exposed to IR+M3541. As previously shown with HeLa cells (Figure 2A), proliferating A549 cells exhibited a large aneuploid/polyploid fraction indicative of defective mitotic transition (Supplementary Figure 2). We then generated metaphase spreads and the chromosome structure was examined by microscopy. In the cells exposed to IR+M3541, an increased number of broken and deformed chromosomes were found compared to vehicle, M3541 or IR alone (Figure 3A). Over 75% of the cells had more than one and 47% more than five aberrations (Figure 3B) compared to 8% in IR only cells.

Next, we performed spectral karyotyping analyses of metaphase chromosomes exposed to vehicle, M3541, IR or IR+M3541. Our results demonstrated substantial increase in chromosomal alterations, including deletions and chromatid breaks (Figure 3C, 3D) with a preponderance of marker chromosomes (Figure 3E), which cannot be defined by their genetic origin due to complex aberrations. Consistent with increased mitotic errors observed in GFP labeled H2B HeLa cells (Figure 2E), M3541 promoted chromosome misalignment in irradiated metaphase A549 cells. Immunofluorescence images showed that the IR+M3541 treatment, in contrast to IR alone, resulted in a higher frequency of chromosomal alignment errors (36% vs. 8%) and high levels of γH2AX signal in metaphase chromosomes (Figure 3F), which can lead to failure in microtubule attachment.

Then, we examined nuclear morphology and the presence of micronuclei in A549 cells exposed to M3541, IR alone or the combination. Treatment with the IR+M3541 combination for 1, 3 and 7 days led to a time-dependent increase of micronuclei positive cells. Moreover, these micronuclei contained large number of unrepaired DSBs, indicated by strong γH2AX staining (Figure 3G). Similar effects were observed in HeLa and HT1080 cancer cells (Supplementary Figure 3A–3D). After 7 days of continuous exposure to M3541, more than 50% of irradiated cells contained one or multiple micronuclei (Figure 3H, 3I) and 25% displayed multiple nuclei (Figure 3J) indicative of aberrant mitoses. These results suggested that M3541 strongly enhances the structural damage in metaphase chromosomes from irradiated cancer cells primarily caused by unrepaired DSBs, leading to abnormal chromosome segregation during mitosis.

### M3541 is a strong enhancer of inflammatory signaling in irradiated cancer cells

**Figure 4:**
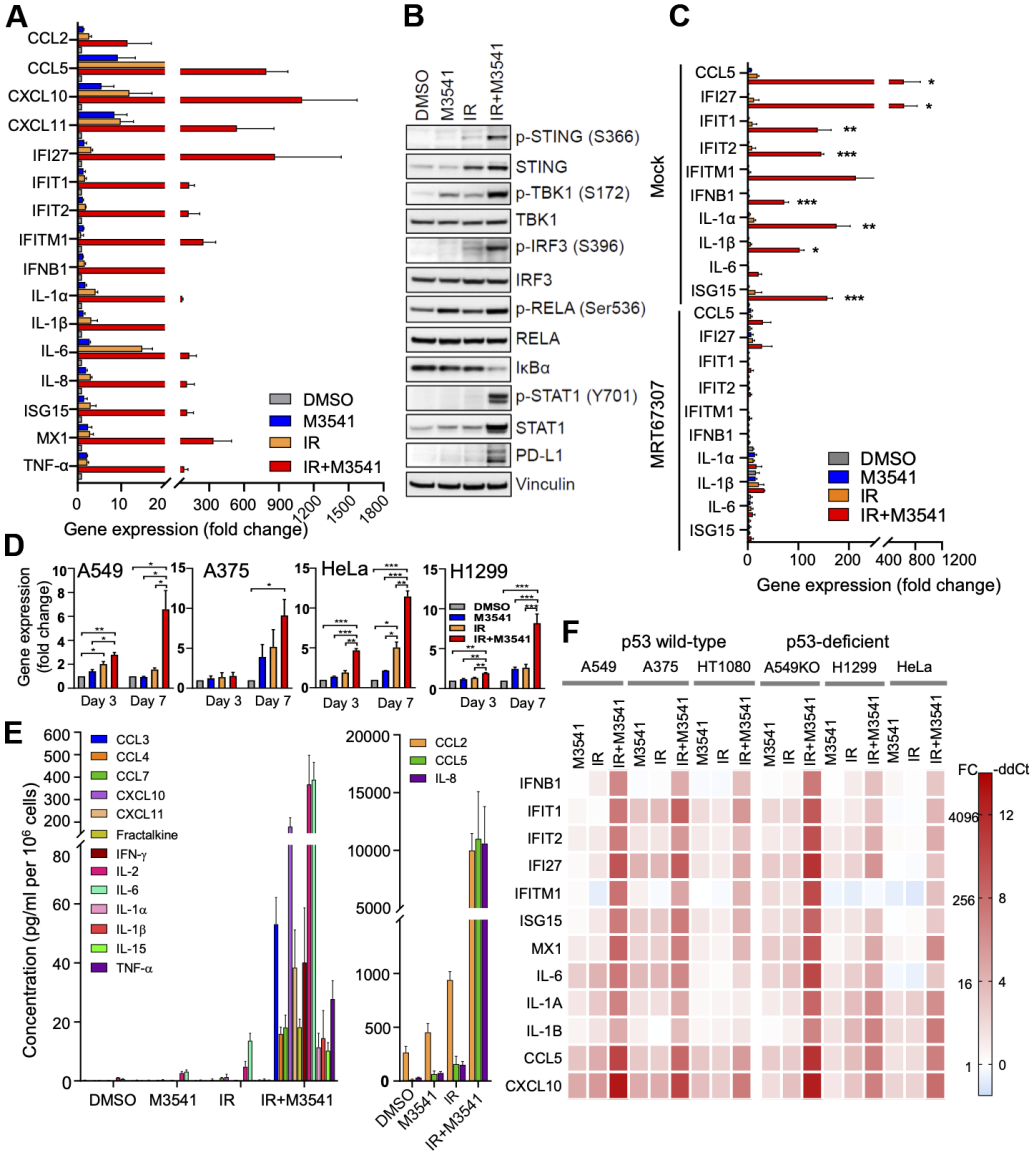
**M3541 is a strong enhancer of inflammatory signaling in irradiated cancer cells.** (**A**) Relative gene expression in A549 cells treated with DMSO, M3541, IR or IR+M3541 for 7 days measured by quantitative PCR. Data are shown as mean±SEM. (**B**) Western blot analysis of cGAS/STING pathway in A549 cells after DMSO, M3541, IR or IR+M3541 treatment for 7 days. (**C**) Relative gene expression in A549 cells treated as above with or without 2 μM TBK1 inhibitor, MRT67307 for 7 days. Data are shown as mean±SEM. (**D**) Relative expression of PD-L1 in A549, A375, HeLa and H1299 cancer cells treated as in (**A**). (**E**) Changes in cytokine levels in the culture media from A549 cells exposed to DMSO, M3541, IR or IR+M3541 for 7 days. Cytokine levels were measured by the MSD technology, normalized to the total cell number and presented as pg/ml per million cells. (**F**) Heatmap of the relative gene expression in 6 cancer cell lines exposed to M3541, IR or IR+M3541 for 7 days.

Previous studies have shown that DNA damaging agents could induce micronucleation and release of DNA in the cytosol, leading to activation of cGAS/STING dependent inflammatory signaling [21–23, 38]. We examined the effect of the ATM inhibitor on inflammatory signaling in irradiated A549 cells *in vitro*. First, we performed quantitative PCR analyses to assess the expression of a panel of Type I Interferon β (IFNβ), interferon-stimulated genes (ISGs) and inflammatory cytokines in proliferating cells exposed to M3541, IR or IR+M3541 (Figure 4A). Compared to IR, combined IR+M3541 treatment strongly enhanced the expression of IFNB1, multiple downstream ISGs (IFIT1, IFIT2, IFITM1, ISG15 and MX1) and inflammatory cytokines/chemokines (CCL2, CCL5, CXCL10, IL-6, IL-8, IL-1A and IL-1B). Exposure to M3541 or IR alone showed elevated expression of a few inflammatory cytokines/chemokines (i.e. CCL5, CXCL10 and CXCL11) but no significant effect on IFNB1 and ISGs expression.

Next, we assessed the effect on the STING pathway in response to the combined IR+M3541 treatment at a protein level. Western blotting revealed that exposure to IR+M3541 markedly increased levels of p-STING, STING, p-TBK1 and p-IRF3, compared to IR alone (Figure 4B). STAT1 phosphorylation, a key downstream target in interferon signaling [22] and total STAT1 levels were robustly elevated in the combination treatment (Figure 4B). Increased levels of total STAT1 have been reported as part of a positive feedback loop to provide sustain expression of IFN responsive genes [39]. In support of this, the addition of the TBK1 inhibitor MRT67307 nearly completely abrogated immune gene upregulation (Figure 4C). It has been reported that Program Cell Death Ligand 1(PD-L1) is upregulated by interferon signaling in tumor microenvironment [40, 41]. In A549 cells, PD-L1 protein expression was dramatically elevated by the combined IR+M3541 treatment compared to all other controls (Figure 4B). This strong PD-L1 upregulation was confirmed in A549 and three other cancer cell lines at a transcriptional level by qPCR (Figure 4D). Adding TBK1 inhibitor MRT67307 prevented PD-L1 upregulation, confirming its dependence on the STING/TBK1 pathway activated by ATM inhibitor in irradiated A549 cells (Supplementary Figure 4A). The time dependence of these effects suggest that it is linked to the progressive increase of micronucleation in the population of irradiated cancer cells undergoing consecutive mitoses with chromosome misalignment and mis-segregation.

Then, we assessed the effect of M3541 on the levels of secreted cytokines in the A549 culture media by the MSD assay. A panel of inflammatory cytokines/chemokines known to be involved in the innate and adaptive immune response was tested after 7 days exposure to DMSO, M3541, IR or IR+M3541 in the A549 cell media and found to be strongly elevated compared to controls (Figure 4E). Moreover, combination treatment with IR and M3541 showed a stimulatory effect on inflammatory signaling in multiple cancer cell lines (Figure 4F). This effect was confirmed with the other ATM inhibitor, M4076 (Supplementary Figure 4). Taken together, our data indicate that continuous ATM inhibition in irradiated cancer cells provides a strong enhancement of the inflammatory signaling and leads to elevated expression and secretion of multiple inflammatory cytokines/chemokines.

### M3541 treatment accelerates NK-cell dependent killing of irradiated cancer cells *in vitro*

**Figure 5:**
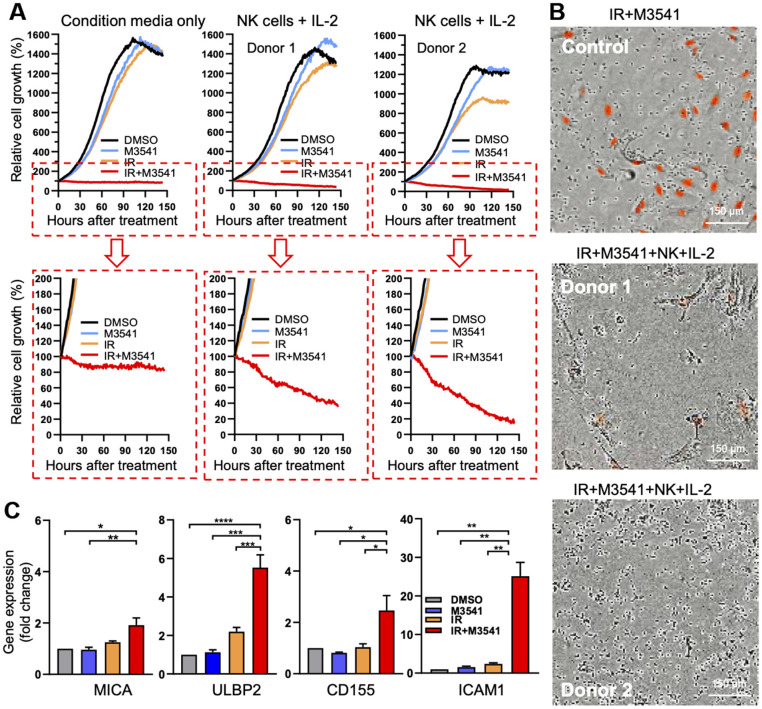
**M3541 treatment accelerates NK-cell dependent killing of irradiated cancer cells *in vitro*.** (**A**) Growth/viability curves of A549 Nuclight Red cells exposed to DMSO, M3541, IR or IR+M3541 for 7 days, reseeded at equal number and co-cultured with NK cells from two healthy donors for 150 hours. Growth curves represent relative change in the number of viable cells (red-fluorescent nuclei) determined by continuous live imaging every 2h by IncuCyte. (**B**) Representative images of the above extracted from IncuCyte time lapse videos. (**C**) Relative expression of NK ligands, MICA, ULBP2, CD155 and ICAM1 in A549 exposed to DMSO, M3541, IR or IR+M3541 for 7 days, measured by quantitative PCR. Data represent mean ± SEM.

To assess whether elevated levels of immune cytokines/chemokines induced by IR+M3541 treatment could enhance innate immune response *in vitro*, we evaluated the cell killing potential of freshly isolated NK cells in co-culture experiments. Proliferating A549 Nuclight Red cells, expressing RFP in the nuclear lamina, were exposed to DMSO, M3514, IR or IR+M3541 for 7 days. Based on our mechanistic findings, this period of treatment was chosen to maximize micronuclei accumulation and STING activation in the surviving cells. Cells from different treatment conditions were then harvested and re-seeded at an equal number under corresponding conditions. NK cells were purified from PBMC of healthy donors and added to re-seeded A549 cells at 10:1 (NK to cancer cells) ratio in the presence or absence of IL-2. Growth/viability curves were derived by continuous live cell imaging (Figure 5A, 5B). While the control cells exposed to DMSO, M3541 or IR continued to proliferate, the cells surviving the IR+M3541 combination were growth arrested and the number was reduced by approximately 20% after the first two days but retained their number for the rest of the observation period. Addition of NK cells significantly decreased the fraction of surviving cells to approximately 40% (Donor 1) and 20% (Donor 2). These results suggested that the combined treatment increased the susceptibility of A549 cells to NK cell killing.

Chemotherapeutic agents have previously been reported to upregulate expression of ligands for NK cell activating receptors thus enhancing NK cell mediated killing [42, 43]. To further probe the mechanisms behind the increased sensitivity of NK cell killing, we examined the expression levels of ligands for NK cell activating receptors including MHC class I-related chain molecules A and B (MICA and MICB), members of the UL-16 binding protein (ULBP) family (ULBP1, ULBP2), CD155 and intercellular adhesion molecule-1 (ICAM1) on irradiated A549 cells exposed to M3541 for 7 days. ICAM1 is known to play a role in the tumor-NK cell interactions, needed for effective NK cytotoxicity [44]. Quantitative PCR analysis revealed that combination treatment with IR+M3541 markedly increased ULBP2, CD155 and ICAM1 expression and slightly MICA expression compared to IR alone (Figure 5C) but did not change MICB and ULBP1 expression (data not shown). These data support the hypothesis that enhanced expression of surface proteins in A549 cells exposed to IR in the presence of M3541 may contribute to the accelerated NK cell killing alongside the elevated secretion of multiple immune related cyto-/chemokines. The specific role of different molecular components warrants further investigation in relevant cellular and animal models, but these results suggest that ATM inhibition could enhance the immunomodulatory effect of radiation in the tumor environment.

## DISCUSSION

ATM kinase is a key regulator of the DNA damage response and has emerged as a potential point for therapeutic intervention in cancer [3, 5, 8, 45]. Several small molecule inhibitors have been described including clinical stage agents in development [30, 45, 46]. However, there are still gaps in our understanding of the detailed mechanisms of response to ATM inhibitors and their therapeutic potential. Using two novel compounds as selective molecular tools, we aimed at dissecting how ATM inhibition modifies cellular responses to radiation and potential therapeutic implications. M3541 and M4076 are small molecules in clinical development from a novel chemical class with high potency and selectivity [30, 47]. They suppress ATM catalytic activity in cancer cells and animal models and have shown strong potentiation of the antitumor activity of ionizing radiation and DSB-inducing chemotherapeutic agents offering possible new combination partners for cancer therapy [30]. Our data show that M3541 and M4076 suppress both key functions of ATM, the cell cycle checkpoint control and prevents the repair of the most toxic DNA lesion. We have also shown that the inability of the mitotic apparatus to handle structurally impaired chromosomes with broken DNA is the main mechanism behind the enhanced antitumor activity by ATM inhibitors.

Ionizing radiation is among the most effective generators of DNA DSBs. Immediately after IR exposure, the MRE11-RAD50-NBS1 (MRN) complex recruits and activates ATM [48] and its key downstream targets, p53 and CHK2 mediating cell cycle checkpoint response to DNA damage [6, 10, 11]. Activated directly by ATM phosphorylation and secondarily by activated CHK2, the master tumor suppressor p53 is stabilized and activates its transcriptional target and pan-CDK inhibitor p21 plus other cell cycle regulators to halt the cell cycle and protect cells from the lethal consequences of replicating and dividing with unrepaired DSBs [12, 49]. M3541 suppressed both p53 and CHK2 branches of ATM downstream signaling to abrogate their G1/S arrest function (Figure 1D, 1E). The cells entered S phase with unrepaired DSBs, leading to activation of the ATR checkpoint and triggered a G2/M arrest via its downstream target CHK1. However, the G2/M arrest was transient and permitted entry into mitosis of cells with unrepaired DSBs. Within several days, the majority of the cells underwent aberrant mitoses, leading to large senescence-like multinucleated cells and cell death (Figure 2A–2D, Supplementary Figure 2). Severe structural abnormalities were detected in the metaphase chromosomes of irradiated cells exposed to ATM inhibitor (Figure 3), preventing proper alignment and segregation at the mitotic spindle. This resulted in the formation of lagging chromosomes at anaphase and massive micronucleation (Figure 2E–2H) yielding strong activation of the STING/TBK1 pathway (Figure 4). Multiple cytokines/chemokines were detected in the culture media, suggesting that ATM inhibition could substantially augment IR-induced inflammatory signaling in cancer cells.

Micronucleation was recently recognized as a key mechanism for cytosolic exposure of chromatin fragments, activation of the cGAS/STING pathway and inflammatory signaling in cancer cells [22, 23]. Our results revealed that in combination with radiation, ATM inhibitors offer a powerful new strategy for enhancement of DSB-mediated micronucleation and the TBK1-dependent inflammatory signaling in cancer cells. Both M3541 and M4076 dramatically augmented pro-inflammatory effects of IR at mRNA and protein levels (Figure 4) and suggested another combination approach to enhancing the antitumor efficacy of radiation via engagement of the immune system. Our *in vitro* co-culture experiments suggested that irradiated cancer cells exposed to M3541 can enhance the killing activity of NK cells from healthy donors likely through high levels of cytokine/chemokine secretion and/or elevated expression of NK ligands (Figure 5). These early experiments hinted that ATM inhibitors could offer a new way for engaging the innate immune response during radiotherapy of local or locally advanced tumors. Also, the elevated PD-L1 expression in cancer cells exposed to the combined IR+M3541 treatment supports combination with a widely used class of immunotherapeutic agents. Two reports published in the course of our work have addressed different immunomodulatory aspects of ATM inhibition [50, 51]. These studies and our data highlight the complexity of the interface between DDR and immune signaling but also opportunities for therapeutic exploration of ATM inhibitors either as single agents or in combination with radiotherapy and other DSB-inducing agents.

Using a selective inhibitor of DNA-PK, which drives the other major DSB repair pathway, non-homologous end joining, we previously showed that p53 plays a critical role in determining the fate of irradiated cancer cells [36]. By suppressing DSB repair in irradiated cancer cells, peposertib (also known as M3814) overactivated ATM signaling and reinforced the p53-dependent checkpoint controls, leading to complete cell cycle arrest in p53 wild-type cells. Cancer cells deficient in p53 function continued to cycle with unrepaired DSBs leading to more severe damage and death via mitotic catastrophe [36]. We recently showed that gross structural defects in chromosomes, misalignment and mis-segregation are the main mechanisms behind the enhanced radiation induced cell death. Importantly, lagging chromosomes during anaphase gave rise to massive micronucleation and activation of the STING/TBK1-dependent inflammatory signaling [32]. These effects of selective DNA-PK inhibition in combination with IR are very similar to the effect of ATM inhibitors described in this manuscript, with one major difference. Inhibition of DNA-PK protected irradiated p53 wild-type cells from imminent death and structural chromosome damage because of the complete p53-dependent cell cycle arrest. ATM inhibitor suppressed the cell cycle checkpoint function of p53 and blurred the p53-dependence of the response to radiation. Both p53 wild-type and p53-deficient cells underwent structural chromosome changes, aberrant mitotic cycles, micronucleation, and activation of inflammatory signaling. Indeed, the inflammatory response was comparable in the parental p53 wild-type and p53-null clone of A549 cells [36] subjected to combined IR+M3541 treatment (Figure 4F).

Our previous reports [32, 36] and the current manuscript highlight the important fact that entry into mitosis with structurally altered chromosomes determines the fate of irradiated cancer cells, regardless of the specific mechanism of intervention in DSB repair. Both DNA-PK and ATM inhibitors prolong the life of radiation induced DSBs and could serve as potent enhancers of inflammatory signaling and cancer cell death, offering potential new agents for combination radiotherapy. The usefulness of these agents would depend on the therapeutic window they afford in cancer patients. In the clinically relevant 6-week xenograft studies, both M3541 and M4076 demonstrated strong efficacy with complete tumor regression in combination with fractionated radiation [30, 35]. Ongoing (NCT04882917) and planned studies will be exploring the clinical potential of M4076.

## Supplementary Material

Supplementary Information

Supplementary Figures
