# Supplementary material for: Selective ATM inhibition augments radiation-induced inflammatory signaling and cancer cell death
Source: Aging (Albany NY). 2023 Jan 17;15(2):492–512. doi: 10.18632/aging.204487 (PMC9925676; doi:10.18632/aging.204487)
Supplement: Supplementary Information [file aging-15-204487-s001.pdf]

SUPPLEMENTARY INFORMATION

Supplementary information appendix

TaqMan probes

Human GAPDH (Hs02786624_g1), IFNB1 (Hs01077958_s1), IFIT1 (Hs03027069_s1), IFIT2 (Hs01922738_s1), IFITM1 (Hs00705137_s1), IFI27 (Hs01086373_g1), ISG15 (Hs01921425_s1), MX1 (Hs00895608_m1), IL6 (Hs00174131_m1), IL8(Hs00174103_m1), IL1A (Hs00174092_m1), IL1B (Hs01555410_m1), CCL2 (Hs00234140_m1), CCL5 (Hs00982282_m1), CXCL10 (Hs00171042_m1), CXCL11 (Hs00171138_m1), TNF (Hs00174128_m1), CD274 (PD-L1, Hs00204257_m1), , MICA (Hs00792195_m1), MICB (Hs00792952_m1), ULBP1 (Hs00360941_m1), ULBP2 (Hs01127964_m1), PVR (CD155, Hs00197846_m1), ICAM1 (Hs00164932_m1).
