# Supplementary material for: Selective ATM inhibition augments radiation-induced inflammatory signaling and cancer cell death
Source: Aging (Albany NY). 2023 Jan 17;15(2):492–512. doi: 10.18632/aging.204487 (PMC9925676; doi:10.18632/aging.204487)
Supplement: Supplementary Figures [file aging-15-204487-s002.pdf]

SUPPLEMENTARY FIGURES

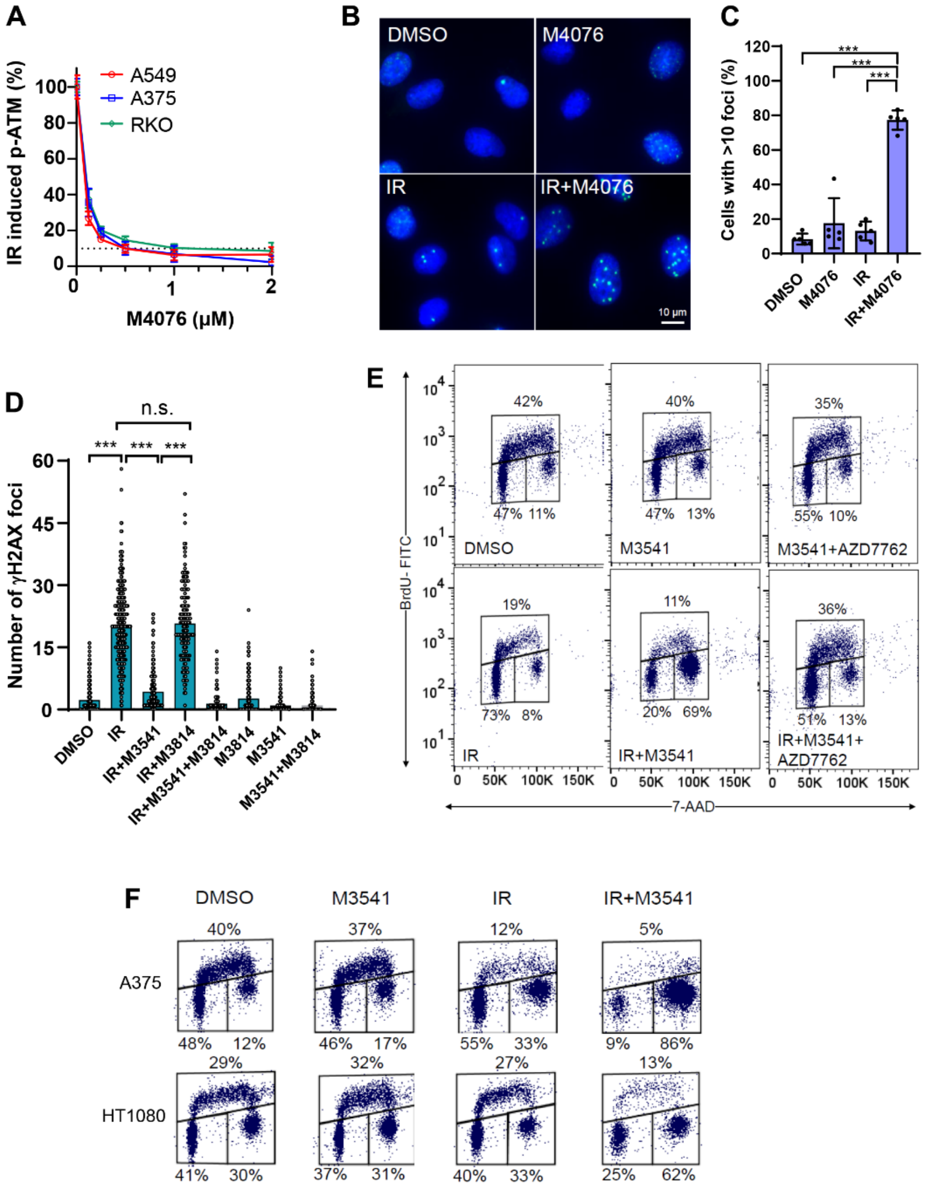

Supplementary Figure 1. M4076 inhibits DSB repair and modifies cellular response to radiation (**A**) Dose-dependent inhibition of ATM autophosphorylation by M4076 in response to radiation. Proliferating A549, A375 and RKO cells were exposed to 5Gy IR to increasing concentrations of M4076 for 1 hour. Cell lysates were collected and ATM phosphorylation at serine 1981 was determined by MSD. The dashed line represents 90% inhibition. (**B**) Analysis of γH2AX foci in A549 cells following exposure to DMSO, 1µM M4076, 5Gy IR, or the combination IR+M4076 at 24 hours post radiation by immunofluorescence. DAPI was used for nuclear counterstain and images were taken at 63x magnification. Scale bar is 10 µm. (**C**) Quantification of γH2AX foci was done by counting the number of cells with >10 foci in images from (**B**) 24 hours post radiation. (**D**) Effect of DNA-PK inhibitor M3814 on IR-induced γH2AX foci in the presence of M3541. A549 cells were exposed to DMSO, M3541 (1 µM), IR (5 Gy) or IR (5 Gy) + M3541 (1 µM) in the presence or absence of M3814 (1 µM) for 30 minutes. Quantification of γH2AX foci per cell nuclei was determined after IR for each indicated condition as in (**C**). (**E**) BrdU cell cycle analyses of A549 cells exposed to DMSO, M3541, IR or IR+M3541 in the presence or absence of CHK1 inhibitor AZD7762 for 24 hours by flow cytometry. The percentage of cells in each phase was calculated and representative data shown. (**F**) BrdU cell cycle analysis of proliferating A375 and HT1080 cells treated with DMSO, M3541 (1 µM), IR (5 Gy) or IR (5 Gy) +M3541 (1 µM) and analyzed as in (**E**).

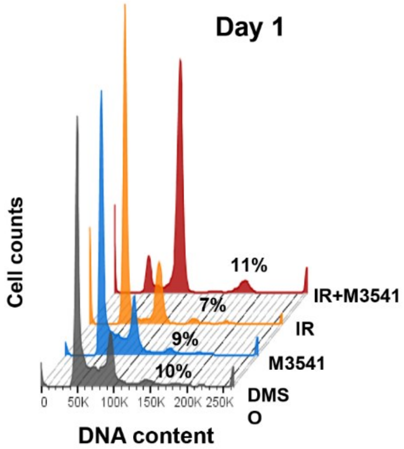

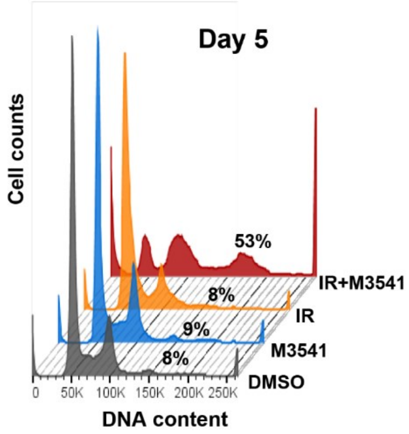

Supplementary Figure 2. M3541 enhances antitumor effect of IR in A549 cells. Cell cycle analysis of 7-AAD labeled A549 cells exposed to DMSO, M3541 (1 μM), IR (5 Gy) or IR (5 Gy)+M3541 (1 μM) on day 1 and day 5. The percentage of cells with > 4N DNA content are indicated.

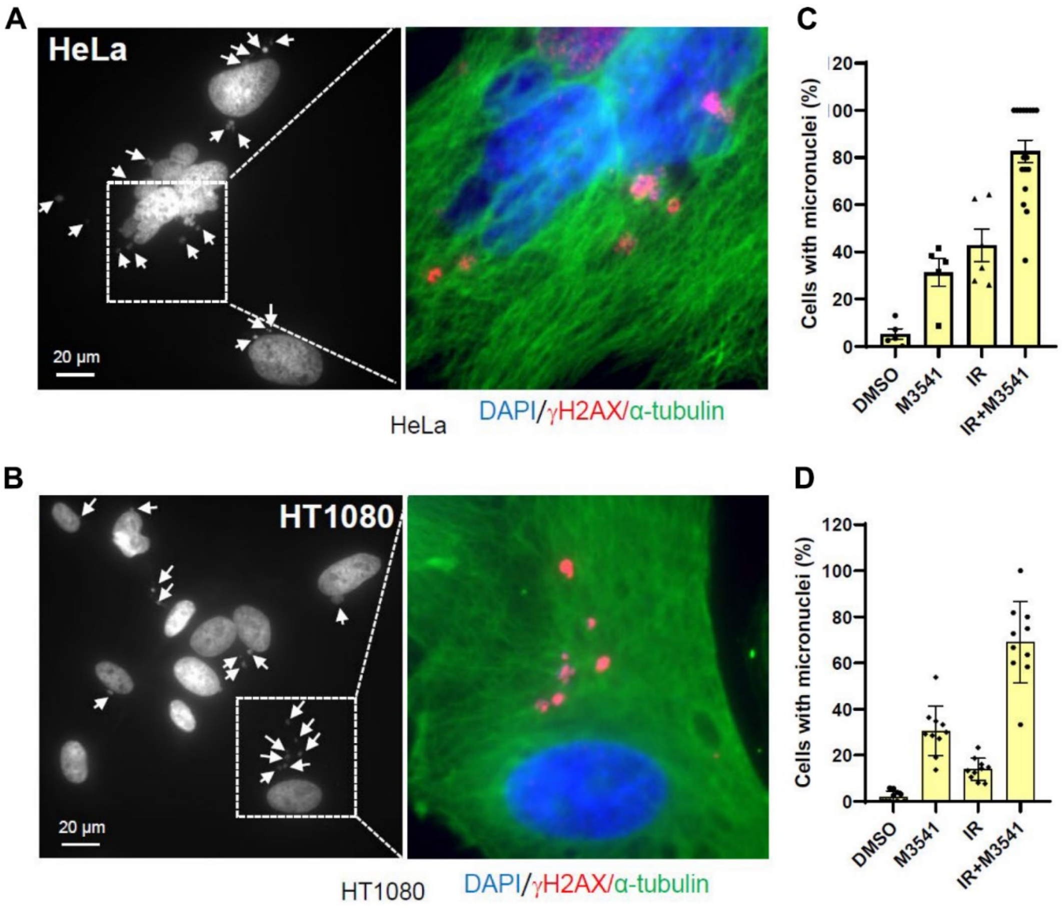

Supplementary Figure 3. M3541 enhanced radiation induced micronucleation in both p53 wild type and p53 deficient cancer cells. Representative immunofluorescence images of DAPI-staining in the p53 dysfunctional HeLa cells (**A**) and the p53 wild type HT1080 cells (**B**) cells exposed to the combination of IR+M3541 for 7 days. Nuclei/micronuclei were stained with DAPI and micronuclei were visualized by co-staining for γH2AX foci and shown in the zoomed area. (**C**) Cells containing >1 micronuclei were counted in immunofluorescent images of HeLa (**C**) and HT1080 cells (**D**) exposed to DMSO, M3541 (1 µM), IR (5 Gy) or IR (5 Gy) +M3541 (1 µM) for 7 days and the percentage of cells with micronuclei is shown as mean±SEM from two independent experiments.

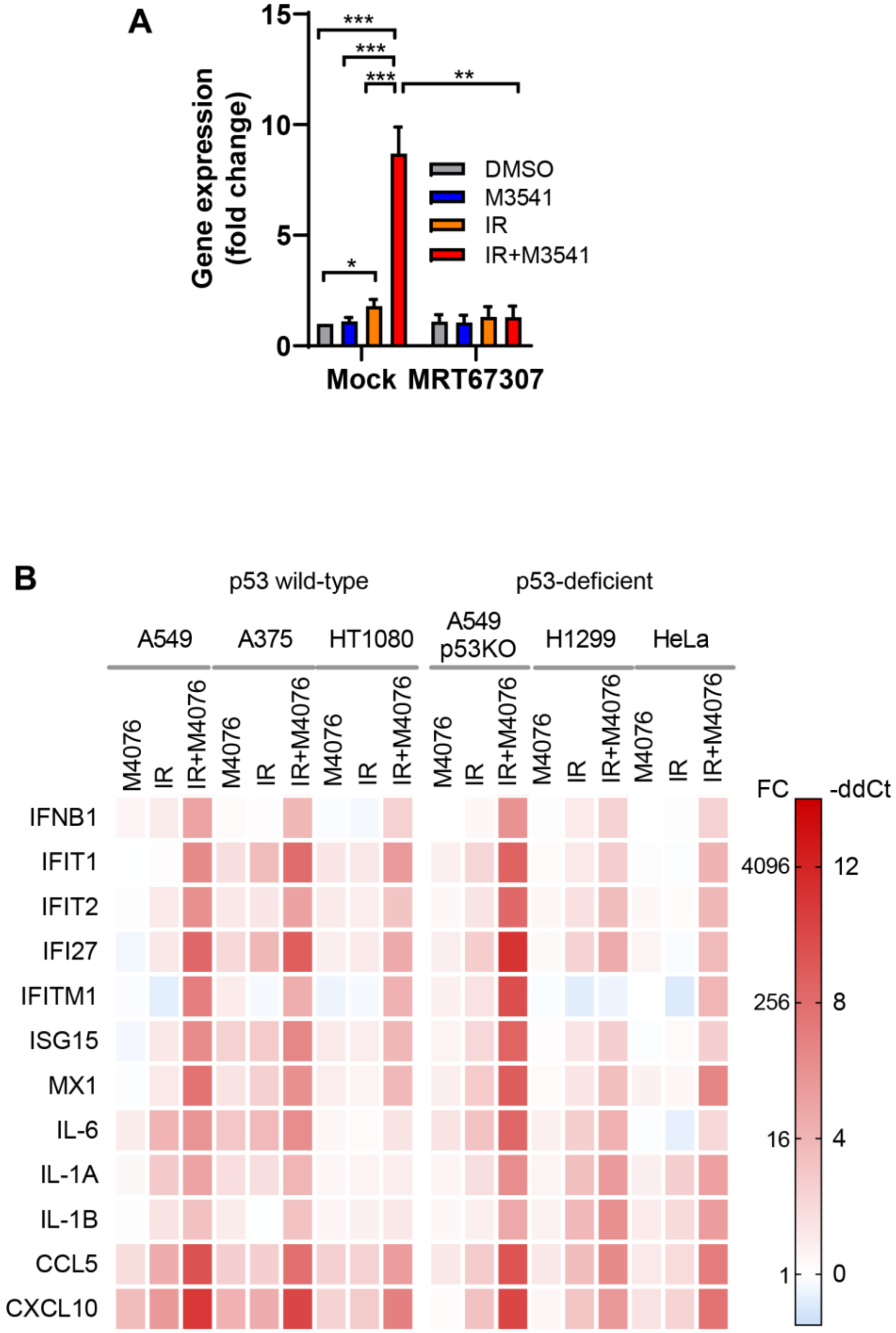

Supplementary Figure 4. M4076 shows similar potentiation of inflammatory signaling as M3541 in multiple cancer cell lines. (**A**) Relative expression of PD-L1 in A549 cells in the presence or absence of 2 μM TBK1 inhibitor, MRT67307 for 7 days. Data are shown as mean ± SEM. (**B**) Heatmap showing the relative expression of a panel of genes known to be activated during inflammatory response in 3 p53 wild-type (A549, A375, HT1080) and 3 p53-deficient (A549 p53-null, H1299, HeLa) cell lines treated with DMSO, M4076 (1 μM), IR (5 Gy) or IR (5 Gy) +M4076 (1 μM) for 7 days. Gene expression was normalized to the DMSO control and shown as fold change.
